# Exploring Burnout: A Study on Psychiatric Nurses in Tunisia

**DOI:** 10.1192/j.eurpsy.2024.702

**Published:** 2024-08-27

**Authors:** H. Khiari, A. Hakiri, M. Bouchendira, R. Ghachem

**Affiliations:** ^1^Psychiatry B; ^2^Psychiatry G, Razi hospital, Mannouba, Tunisia

## Abstract

**Introduction:**

Burnout, marked by persistent workplace stress without effective management, is particularly pertinent for psychiatry nurses, considering the nature of their work environment and its potential impact on the quality of care they deliver.

**Objectives:**

To assess the prevalence of burnout among psychiatric nurses and to identify the socio-demographic and clinical factors associated with it.

**Methods:**

Cross-sectional, descriptive, and analytical study conducted over the course of one month from October 11^th^ to November 8^th^ 2023. Participants included were psychiatric nurses working in Razi Hospital, Tunisia. We collected data using pre-established questionnaire which included socio-demographic and clinical data of the participants. The assessment of Burnout was conducted using the Maslach Burnout Inventory (MBI), validated in Arabic. Statistical analysis was performed using the Statistical Package for Social Sciences (SPSS) in its 25th version.

**Results:**

We collected data from 55 nurses working in Razi psychiatry hospital during the time of the study. Among them, 80% (n=44) were female. Their median age was 35 (Min=25, Max=62). Most of participants were married (81.8%, n=45) and 70.9 (n=39) had kids. In our sample, 5.5% (n=3) and 23.6% (n=13) had respectively personal psychiatric and somatic history. Some addictive behaviors were identified among our participants, especially smoking (14.5%, n=379) and alcohol use (3.6%, n=2).

Regarding working conditions, 81.8% (n=45) were assigned shift work. They worked in the men’s ward (43.6%, n=24), the women’s ward (34.5%, n=19), or in both (21.8%, n=12). Furthermore, 45.5% (n=25) reported witnessing a suicide attempt during their work, and 74.5% (n=41) were victims of aggression, primarily by patients (82.5%, n=33). Sixty percent (n=33) said expressed a desire to transfer.

According to the MBI, 49.1% (n=27) had high emotional exhaustion, 27.3% (n=15) had high depersonalization and 67.3% (n=37) had low personal accomplishment.

A significant association was found between low personal accomplishment and the desire to transfer to another department (p=0.026). No further links were found with other clinical data.

**Conclusions:**

Our findings provide a thorough examination of burnout among psychiatric nursing professionals, underscoring the critical need for specific interventions tailored to their unique challenges.

**Disclosure of Interest:**

None Declared

